# A Beautiful Premium

**Published:** 1876-12

**Authors:** 


Mr. M. J. Richardson, the proprietor of a valuable monthly paper called *The Housekeeper's Companion*, has purchased a fine picture entitled "Forgiveness," a copy of which he proposes to send to each subscriber to his paper who remits him seventy-five cents. The subscription price of *The Housekeeper's Companion* is 50 cents a year. It is, we believe, the only domestic journal of real value which has ever appeared at this price. It is admirable in matter and style, and has several features which will commend it to the majority of housekeepers. It has, moreover, arranged with an able writer on Foods to supply an article each month on the very marked improvements which are being made in food substances at home and abroad. In these articles attention will be given to the very efficient work now being performed by the Health Food Company and others, and the errors of Graham and others will be pointed out. We recognize in this publication a very valuable and useful adjunct to HALL'S JOURNAL, and heartily commend it to our readers. For the sum of $2.25, we will mail both publications one year to any person in the United States, and will also send the premium to all such subscribers as remit 10 cents in addition for postage.

"Forgiveness" is an oil chromo, 16 by 20 inches in size, and finely executed. It represents the Magdalena mentioned in the 27th chapter of Luke, after she is bade to "go and sin no more." It is a beautiful woman whom the rabble have taken and dragged before Jesus;

> "Bringing her within the temple with her head and bosom bare,
> No disguise to hide her blushes, save her vail of golden hair."

We understand that this work of art has usually sold at $5. Mr. Richardson now controls it, and will use it as a premium, as we have said. We have one in our office, and the universal verdict is that it would adorn parlor or picture gallery in the land. We invite our friends to call and examine it.

